# A case report of histiocytic sarcoma mimicking acute pericarditis

**DOI:** 10.1093/ehjcr/ytaf594

**Published:** 2025-12-04

**Authors:** Mattia Corianò, Niccolò Siviero, Nicola Gasparetto

**Affiliations:** Cardiology Unit, Department of Cardiac, Thoracic, Vascular Sciences and Public Health, University of Padua, Via N. Giustiniani 2, 35128 Padua, Italy; Cardiology Unit, Department of Cardiac, Thoracic, Vascular Sciences and Public Health, University of Padua, Via N. Giustiniani 2, 35128 Padua, Italy; Cardiology Unit, Ospedale Ca’ Foncello, Viale Vittorio Veneto 10, 31100 Treviso, Italy

**Keywords:** Cardiac masses, Cardiac tumours, Histiocytic sarcoma, Case report

## Abstract

**Background:**

Pericardial masses are rare disease requiring incremental diagnostic workout to differentiate benignant from malignant lesions. No solid evidence exists regarding their management, and their treatment requires a case-by-case evaluation.

**Case summary:**

A 54-year-old man was diagnosed with a pericardial mass. After performing cardiac magnetic resonance and computed tomography, a high suspicion of malignancy was raised. Positron emission tomography and pericardial biopsy confirmed the presence of a primary histiocytic sarcoma. After multidisciplinary evaluation, the mass was considered not suitable for surgical removal, and a neoadjuvant chemotherapy strategy was started. The first chemotherapy regimen—consisting of cyclophosphamide, doxorubicin, etoposide, vincristine, and methylprednisolone—was not effective. Therefore, a second, more aggressive, regimen consisting of cladribine, cytarabine, granulocyte-colony stimulating factor, and mitoxantrone was started. Following the first cycle, the patient developed bone marrow aplasia and septic shock leading to the exitus.

**Discussion:**

Histiocytic sarcomas primarily involving the heart are extremely rare. Surgical excision represents the first therapeutic choice when feasible. When not pursuable, a neoadjuvant therapy is preferred, although no consensus exists regarding the chemotherapy regimen.

Learning points

- Histiocytic sarcoma is a rare haematopoietic neoplasm derived from non-Langerhans histiocytic cells, and no standardized therapy protocols are currently available.
- A multidisciplinary approach involving cardiologists, radiologists, oncologists, and pathologists is the optimal strategy for tailoring therapy to individual patients affected by histiocytic sarcoma.

## Introduction

Cardiac masses comprise a broad spectrum of differential diagnoses, ranging from benign to malignant lesions.^1^ Their clinical manifestations are diverse and may include chest pain, arrhythmias, heart failure, or even myocardial infarction.^2^ Prompt detection and accurate classification are crucial, especially in the context of cardiac malignancies such as sarcomas, which are highly aggressive and associated with poor outcome.^3^ In this report, we present a case of histiocytic sarcoma (HS) that initially mimicked acute pericarditis.

## Summary figure

**Figure:**
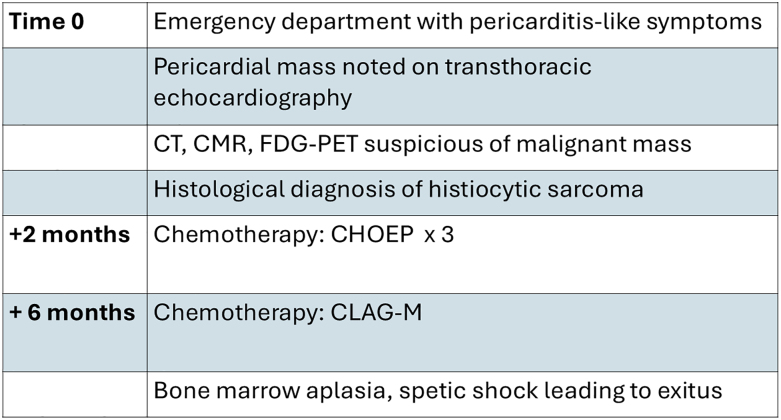


## Case presentation

A 54-year-old man presented to the emergency department with a 3-day history of chest pain, worsened by deep inspiration and the supine position, and relieved by sitting upright. He had no significant past medical history but reported cough, fatigue, sweating, and fever in the previous days.

**Figure 1:**
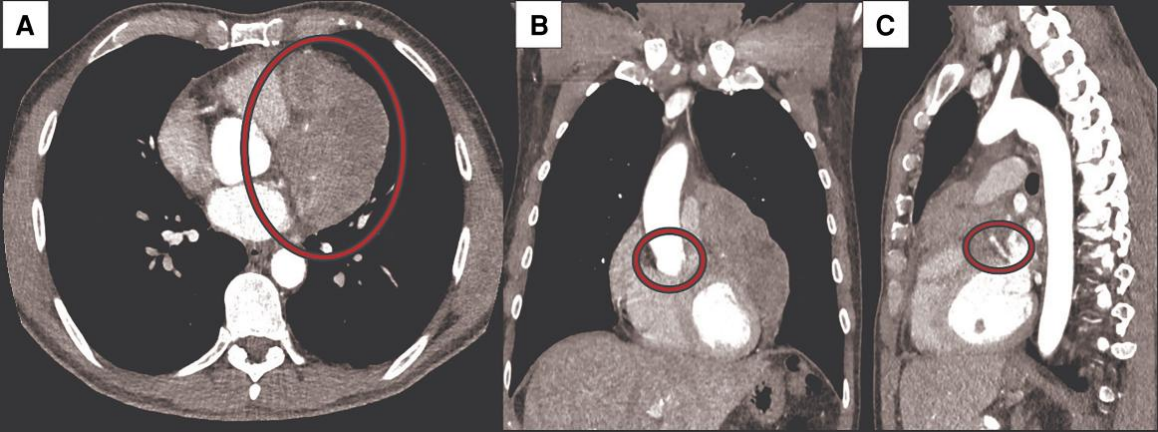
Computed tomography images of the pericardial mass. The mass has an inhomogeneous surface involving the anterolateral wall of the left ventricle (*A*), the right ventricle outflow tract (*B*), and encircling the left anterior descending coronary artery (*C*).

**Figure 2:**
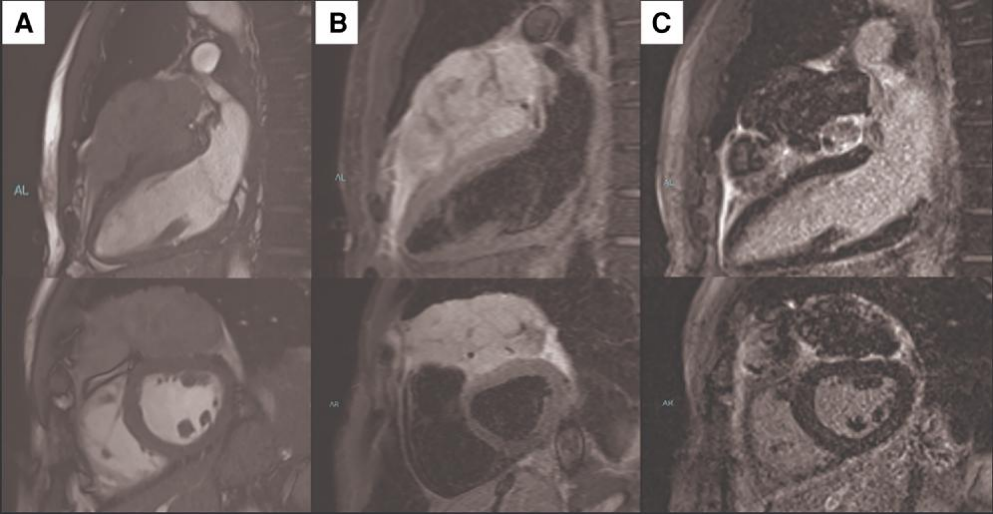
Cardiac magnetic resonance images of the mass. Cine images reveal a large inhomogeneous slightly hypointense mass within the two pericardial layers (*A*). Black-blood T2w images revealed a hyperintensity of the mass and of the pericardial layers (*B*). Late gadolinium enhanced images show a heterogeneous contrast enhancement of the mass and of the pericardial layers (*C*). The mass does not appear to infiltrate the myocardium.

**Figure 3:**
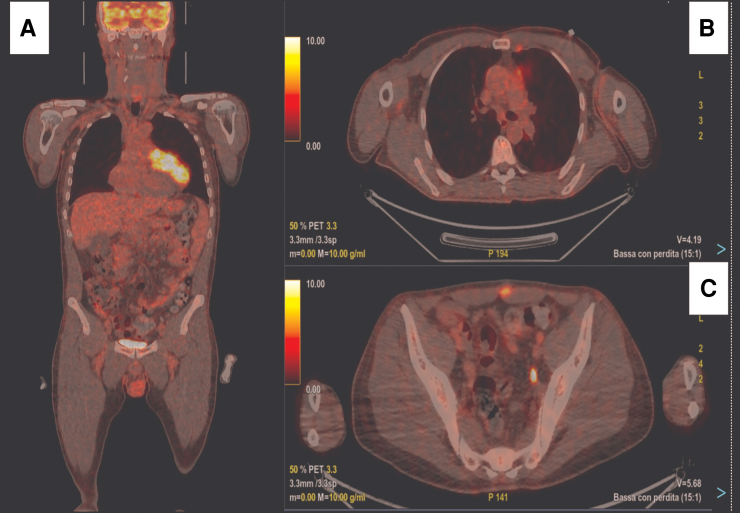
18-Fluoro-deoxy-glucose positron emission tomography. The mass has an increased metabolic activity (maximum standardized uptake value 14.42) within the mass (*A*). Two other areas of increased metabolic activity are in the sternal region (*B*) and hypogastric region (*C*).

**Figure 4:**
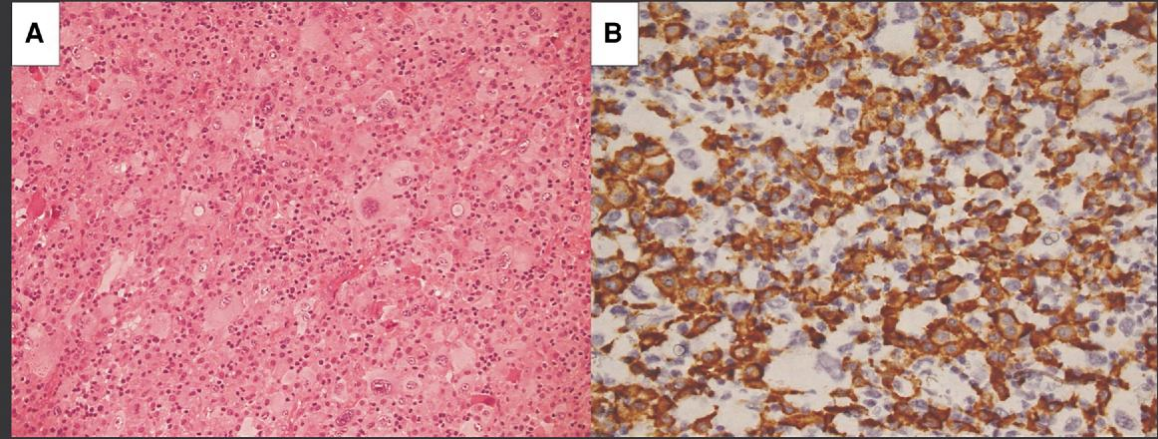
Histopathological analysis of the hypogastric lymph node. The haematoxylin–eosin preparation shows proliferation of histiocytic elements with polymorphic nuclei, prominent evidence of mitotic activity, and moderate mitotic activity (*B*). The immunohistochemical analysis reveals CD163+, CD68+, S100+, CD4 +, and CD1a−, in line with histiocytic sarcoma.

On clinical evaluation, he was asymptomatic at rest, afebrile, with normal vital signs (heart rate 98 b.p.m., blood pressure 129/89 mmHg, oxygen saturation 96%), and a lump in the suprapubic region. The electrocardiogram showed sinus tachycardia, right bundle branch block, and isolated nonspecific T-wave abnormalities in V1–V2. Laboratory tests showed elevated C-reactive protein (15 mg/dL, reference 0–0.50 mg/dL) with normal troponin T levels. Suspecting acute pericarditis, the patient was referred for a cardiologic evaluation. Transthoracic echocardiography revealed persistent mild circumferential pericardial effusion and an intrapericardial mass measuring 4.5 cm in diameter (*Video 1*). The mass involved the anterolateral wall of the left ventricle (LV) and the right ventricular outflow tract (RVOT) without causing haemodynamic compromise. Further evaluation with contrast-enhanced chest computed tomography (CT) identified an inhomogeneous lesion within the pericardium, measuring 10 × 9 × 7 cm. The mass encircled the LV, left atrial appendage, RVOT, left main coronary artery, and proximal segment of the left anterior descending (LAD) artery (*Figure 1*). Cardiac magnetic resonance (CMR) was performed, revealing a solid, multilobulated mass, encircling, but not infiltrating, the cardiac structures. It exhibited hypointensity on T1-weighted images and hyperintensity on T2-weighted images, with inhomogeneous late gadolinium enhancement (*Figure 2*). Subsequently, an 18-fluoro-deoxy-glucose positron emission tomography (18 FDG-PET)/CT scan was performed, showing increased metabolic activity [maximum standardized uptake value (SUV) max 14.42] within the mass. Additionally, a subcutaneous nodule in the hypogastric region and two lymph nodes in the sternal region demonstrated elevated FDG uptake (*Figure 3*). A biopsy of the subcutaneous nodule was performed, and histopathological analysis demonstrated the proliferation of histiocytic elements with polymorphic nuclei, prominent evidence of mitotic activity, and moderate mitotic activity (*Figure 4*). Immunohistochemical analysis showed positivity for CD163 and CD68, suggesting a histiocytic origin, along with S100+, CD4 +, and CD1a−. Molecular analysis identified BRAF gene mutation and clonality in the immunoglobulin heavy chain and T-cell receptor genes. All these findings were diagnostic for HS.

For staging purposes, a bone marrow biopsy was conducted, revealing no bone marrow involvement. After discussion in a multidisciplinary team, surgical removal was excluded due to the impossibility of radically removing the mass. Therefore, a conservative approach with five cycles of CHOEP (cyclophosphamide, doxorubicin, etoposide, vincristine, methylprednisolone) was initiated. After three chemotherapy cycles, a FDG-PET/CT scan was repeated, showing a slight reduction in tracer uptake in the pericardial mass (SUV 12.3) and no further uptake in hypogastric and thoracic regions. However, CT imaging revealed a slight increase in the size of the pericardial mass (11 × 9 × 9 cm) and splenomegaly. Additionally, the presence of multiple new cutaneous lesions of unclear aetiology was noted, although they were not further biopsied. Due to the poor response to chemotherapy, the case was re-evaluated by the heart team. A thoracotomy biopsy was performed, confirming the diagnosis of HS. The patient was subsequently admitted, and a new chemotherapy regimen with CLAG-M (cladribine, cytarabine, granulocyte-colony stimulating factor, mitoxantrone) was started. Following the first cycle of CLAG-M, the patient developed bone marrow aplasia and septic shock requiring vasopressors. A progressive clinical deterioration followed, leading to the exitus.

## Discussion

To the best of our knowledge, this represents the second reported case of primary HS involving the pericardium, and the first one with comprehensive description of diagnosis, therapeutic approach, and follow-up.

Primary cardiac tumours (PCTs) are rare neoplasms, occurring at a rate of 1380 per 100 million individuals annually, with up to 90% presenting a benign histology.^1^ Cardiac tumours may be symptomatic or discovered incidentally during investigations for unrelated conditions. Clinical manifestations can include systemic symptoms such as fever, arthralgia, weight loss, and fatigue. Additionally, PCTs may cause cardiac symptoms due to mass effects that interfere with myocardial function or blood flow, potentially leading to arrhythmias, valvular insufficiency, and pericardial effusion.^2^

The diagnosis of PCTs involves multimodal imaging techniques aimed at confirming the presence of a cardiac mass, determining its location within the cardiac structures, and, when possible, distinguishing between malignant and benign tumours. Trans-thoracic echocardiography (TTE) is the first-line diagnostic tool, useful for defining the tumour’s location and its haemodynamic impact.^1^

Computed tomography imaging offers superior morphological characterization and aids in identifying the tumour’s primary location and staging malignant lesions. Cardiac magnetic resonance imaging is the preferred technique for tissue characterization, as it can detect fatty infiltration, calcification, haemorrhage, cellularity, and vascularity of the mass.^4^ Positron emission tomography assists in differentiating between malignant and benign lesions and in staging malignant tumours.^5^

The therapeutic approach depends on the specific tumour type. For cardiac sarcomas, complete surgical resection is the treatment of choice. When complete resection is not feasible, surgical debulking may be performed to relieve symptoms. In some cases, neoadjuvant chemotherapy can improve survival in selected patients.^3^ For cardiac lymphomas, treatment typically consists of anthracycline-based chemotherapy in combination with the monoclonal anti-CD20 antibody rituximab.^6^

Histiocytic sarcoma is a haematological malignancy that arises from non-Langerhans histiocytic cells, with an incidence of 0.17/1 000 000 per year. It is more common in males, with a median age at diagnosis of ∼63 years. Histiocytic sarcoma can present as an isolated malignancy, or be associated with other haematological tumours, most commonly non-Hodgkin lymphomas, acute myeloid leukaemia, and chronic lymphocytic leukaemia.^7,8^

Histiocytic sarcoma may involve a single organ, causing symptoms related to local invasion and compression, or it may present as a diffuse form with multiorgan involvement. It typically affects connective tissues, lymph nodes, and the skin, although other sites such as the respiratory system, nervous system, gastrointestinal tract, bone marrow, spleen, and reticuloendothelial system can also be involved.^7,8^ Primary cardiac involvement is extremely rare, with only two cases reported to date.^9,10^

Histopathological analysis represents the diagnostic gold standard. Differential diagnoses include Langerhans cell histiocytosis and Rosai–Dorfman disease, both of which show positivity for S-100. However, the former differs from HS by its positivity for CD1a and CD207 (langerin), while the latter differs by the absence of the BRAF V600E mutation. In addition, certain histological features, such as emperipolesis, can assist in the differential diagnosis.^7^

The prognosis for cardiac sarcomas is generally poor, with a median survival of ∼2.5 months for angiosarcoma and 6 and 12 months for leiomyosarcoma and rhabdomyosarcoma, respectively.^4,5^ Radical surgical excision is considered the treatment of choice; however, when complete resection is not feasible, neoadjuvant chemotherapy has been shown to improve survival and increase the likelihood of successful tumour removal.^8^

Treatment of cardiac HS has only been described once previously by Thattassery *et al*.^10^ In that case, the tumour presented as a left ventricular mass. The patient underwent partial excision followed by adjuvant chemotherapy; however, due to poor response to treatment and disease progression, the patient received adjuvant radiation therapy along with several cycles of chemotherapy, which were complicated by pancytopenia and persistent fever. The patient eventually underwent palliative radiation therapy and died 7 months after receiving a diagnosis.

In contrast, our case differed in several key aspects. Radical surgery was not feasible because of the tumour’s location, its involvement of critical cardiac structures, and the presence of lymph node metastases. Based on the haematologic origin of the tumour cells and the established effectiveness of CHOEP-21 in haematologic malignancies, a CHOEP-21 regimen was initially selected after multidisciplinary discussion and an extensive literature research. We found that in a case series of four patients with non-cardiac forms, three were treated with a CHOEP regimen; the same treatment regimen was reported in another case series involving two patients with vertebral involvement.^11,12^

Due to lack of response to CHOEP-21 after three cycles, a biopsy of the pericardial mass was performed, confirming the diagnosis of HS. According to the literature, CLAG-M has been successfully used as a second-line therapy in cases where lymphoma-directed regimens were ineffective.^13^ Therefore, this regimen was initiated. However, despite this intervention, the patient’s condition progressively deteriorated, ultimately leading to death.

## Lead author biography


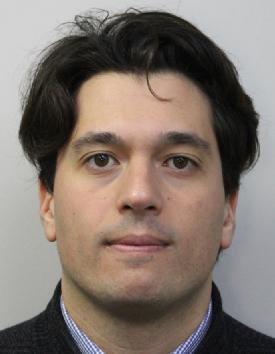


Mattia Corianò completed his training in cardiology at the University of Padua. Currently, he is a PhD student at the University of Padua and a Chain Florey Fellow at Imperial College London. His clinical interests include heart failure, CMR, and heart transplantation. His research focuses on the application of artificial intelligence in cardiology. During his training, Mattia Corianò served as a specialty trainee at the Ca Foncello Hospital in Treviso, working in the intensive care unit and echo lab.

## Data Availability

The data supporting the findings of this study are available from the corresponding author upon reasonable request.
